# Influence of relatively short-term culture on adult porcine islets for xenotransplantation

**DOI:** 10.1038/s41598-024-62570-6

**Published:** 2024-05-21

**Authors:** Naoaki Sakata, Gumpei Yoshimatsu, Ryo Kawakami, Shohta Kodama

**Affiliations:** 1 Department of Regenerative Medicine and Transplantation, Faculty of Medicine, Fukuoka University, 7-45-1 Nanakuma, Jonan-ku, Fukuoka 814-0180 Japan; 2 Center for Regenerative Medicine, Fukuoka University Hospital, Fukuoka, Japan

**Keywords:** Islet transplantation, Xenotransplantation, Pig, Culture, Diabetes mellitus, Translational research, Cell culture, Cell transplantation

## Abstract

Porcine islet xenotransplantation is a promising therapy for severe diabetes mellitus. Maintenance of the quality and quantity of porcine islets is important for the success of this treatment. Here, we aimed to elucidate the influence of relatively short-term (14 days) culture on adult porcine islets isolated from three micro-minipigs (P111, P112 and P121). Morphological characteristics of islets changed little after 14 days of culture. The viability of cultured islets was also maintained at a high level (> 80%). Furthermore, cultured islets exhibited similar glucose-stimulated insulin secretion and insulin content at Day 14 were preserved comparing with Day 1, while the expressions of *Ins*, *Gcg* and *Sst* were attenuated at Day 14. Xenotransplantation using diabetic nude mice showed no normalization of blood glucose but increased levels of plasma porcine C-peptide after the transplantation of 14 day cultured porcine islets. Histological assessment revealed that relatively short-term cultured porcine islets were successfully engrafted 56 days following transplantation. These data show that relatively short-term culture did not impair the quality of adult porcine islets in regard to function, morphology, and viability. Prevention of impairment of gene correlated with endocrine hormone is warranted for further improvement.

## Introduction

Islet transplantation is a promising cellular replacement therapy for patients in severe diabetes with poor glucose control. This treatment is limited by the lack of a human donor pool^1^, and therefore, establishment of alternative donor sources is warranted. Among the various candidates, the adult pig is considered one of the potential donors. The pig has merits in the easiness to breed and produce animals of an appropriate size and number. Furthermore, porcine insulin agents were previously used in clinical practice before the development of recombinant human insulin, indicating porcine insulin can be used to treat patients with diabetes. While porcine islet transplantation harbors some limitations in immunity, correlated with porcine-specific carbohydrate antigens [such as galactose-α-1,3-galactose (α-Gal) and *N*-glycolyl neuraminic acid (Neu5Gc)]^2–6^ and zoonosis (including porcine endogenous retrovirus)^7,8^, recent progress of gene-editing technology enables investigators to overcome these limitations^9,10^. Therefore, clinical porcine islet xenotransplantation may be feasible in the near future.

For the promotion of porcine islet xenotransplantation, the provision of porcine islets with quality assurance is one of the important challenges. Elucidation of the acceptable culturing timespan for porcine islets is one of the critical approaches for the provision. In a previous study, we assessed the influence of long-term (28 days) culture of porcine islets and revealed that glucose-stimulated insulin secretion (GSIS) significantly declined after culture^11^. This research indicated that 28 days of culture was too long for preserving the quality of islets, and a shorter span was recommended.

In this study, we aimed to elucidate the influence of relatively short-term culture (14 days) on the characteristic of adult porcine islets.

## Results

### Relatively short-term culture preserved the morphology and viability of adult porcine islets

**Figure 1:**
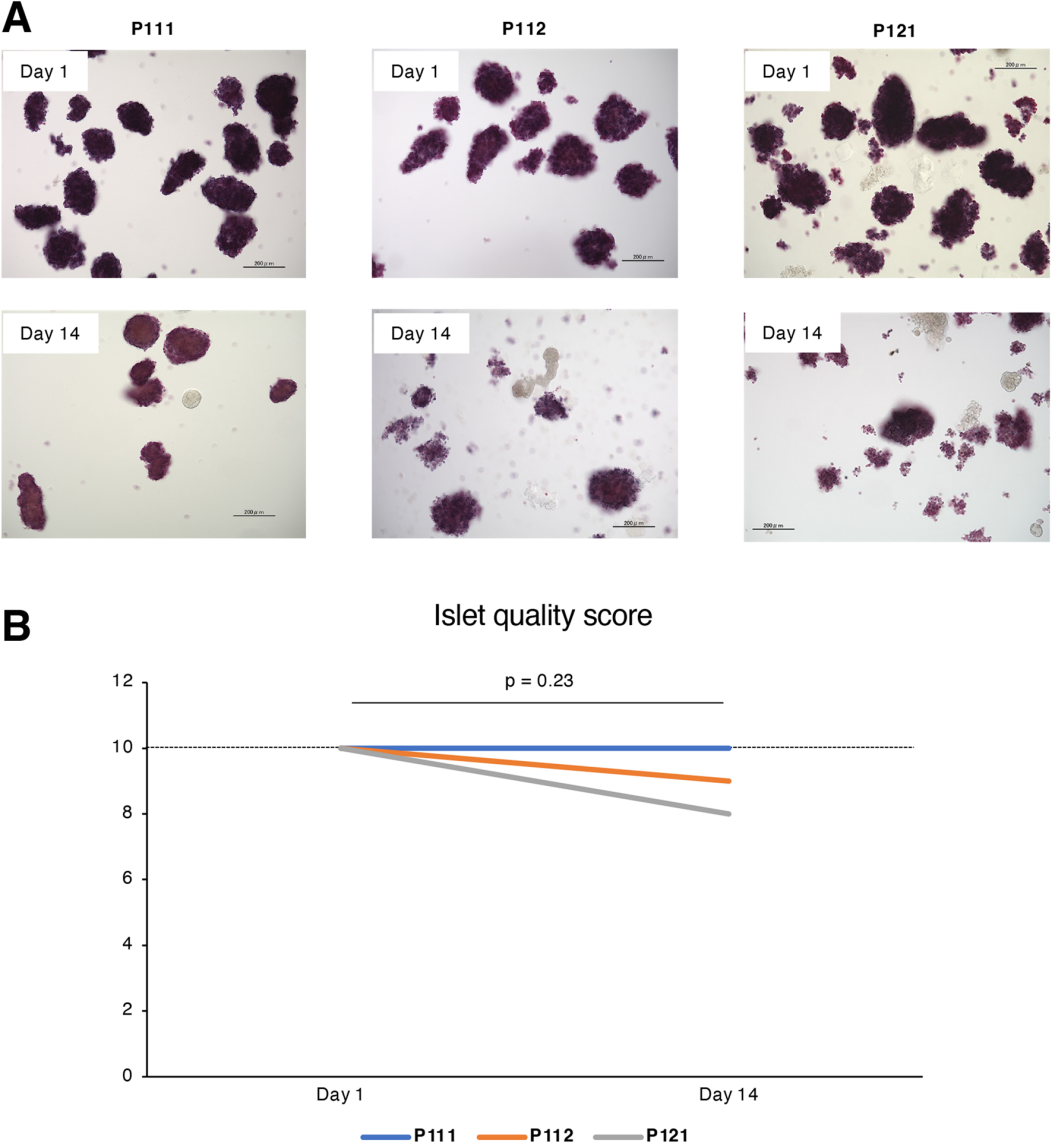
Morphology of relatively short-term cultured porcine islets. (**A**) Dithizone-stained porcine islets, isolated from pigs P111, P112 and P121, on days 1 (upper) and 14 (lower) of culture. Scale bar: 200 µm. (**B**) Change of islet quality score of cultured islets be-tween days 1 and 14.

We first determined the influence of relatively short-term culture on the morphology of porcine islets. Most of the islets obtained from pig P111 were oval or round in shape, with smooth surfaces on Day 1, and these morphological characteristics had changed little on Day 14 (Fig. 1A). Islets from pig P112 showed similar morphological characteristics on Day 1. While some of the islets had a rough surface on Day 14, most showed a preservation of morphology (Fig. 1A). Islets from pig P121 were also oval and round shape with a smooth surface on Day 1, similar to the other cell isolations. On Day 14, the surface of the islets tended to be rough and single cell numbers were increased. Islet shape was preserved (Fig. 1A). The islet quality score was maintained at a high level and was not significantly changed between Day 1 and Day 14 for each isolation (P111: 10 to 10; P112: 10 to 9; P121: 10 to 8; P = 0.23) (Fig. 1B; Table 1).

**Table 1:** Characteristics of the donor microminipigs and isolated islets.

	P111	P112	P121
Body weight (kg)	29.2	28.2	26.3
Trimmed pancreas weight (g)	96	69	60
Warm ischemic time (min)	0	0	0
Cold ischemic time (min)	110	122	159
Islet yields after purification (IEQs)	87,618	109,349	187,542
Islet yields after purification (islet numbers)	116,000	118,000	173,548
IEQ/islet number after purification	0.76	0.93	1.08
Purity after purification on Day 1 (%)	95	90	95
Islet quality score after purification on Day 1 (0–10)	10	10	10
Purity after purification on Day 14 (%)	95	95	90
*Islet quality score after purification on Day 14 (0–10)	10	9	8

*Islet quality score (0–10): sum of the scores in islet shape, surface, damage, the number of single cells in the culture medium and the diameters of the islets.

Islet shape: flat/planar at 0, in between at 1, spherical at 2.

Border: irregular at 0, in between at 1, well-rounded at 2.

Integrity: fragmented at 0, in between at 1, solid/compact at 2.

The number of single cells: many at 0, a few at 1, almost none at 2.

The diameters of the islets: all islets < 100 µm at 0, a few islets > 200 µm at 1, over 10% of islets > 200 µm at 2.

**Figure 2:**
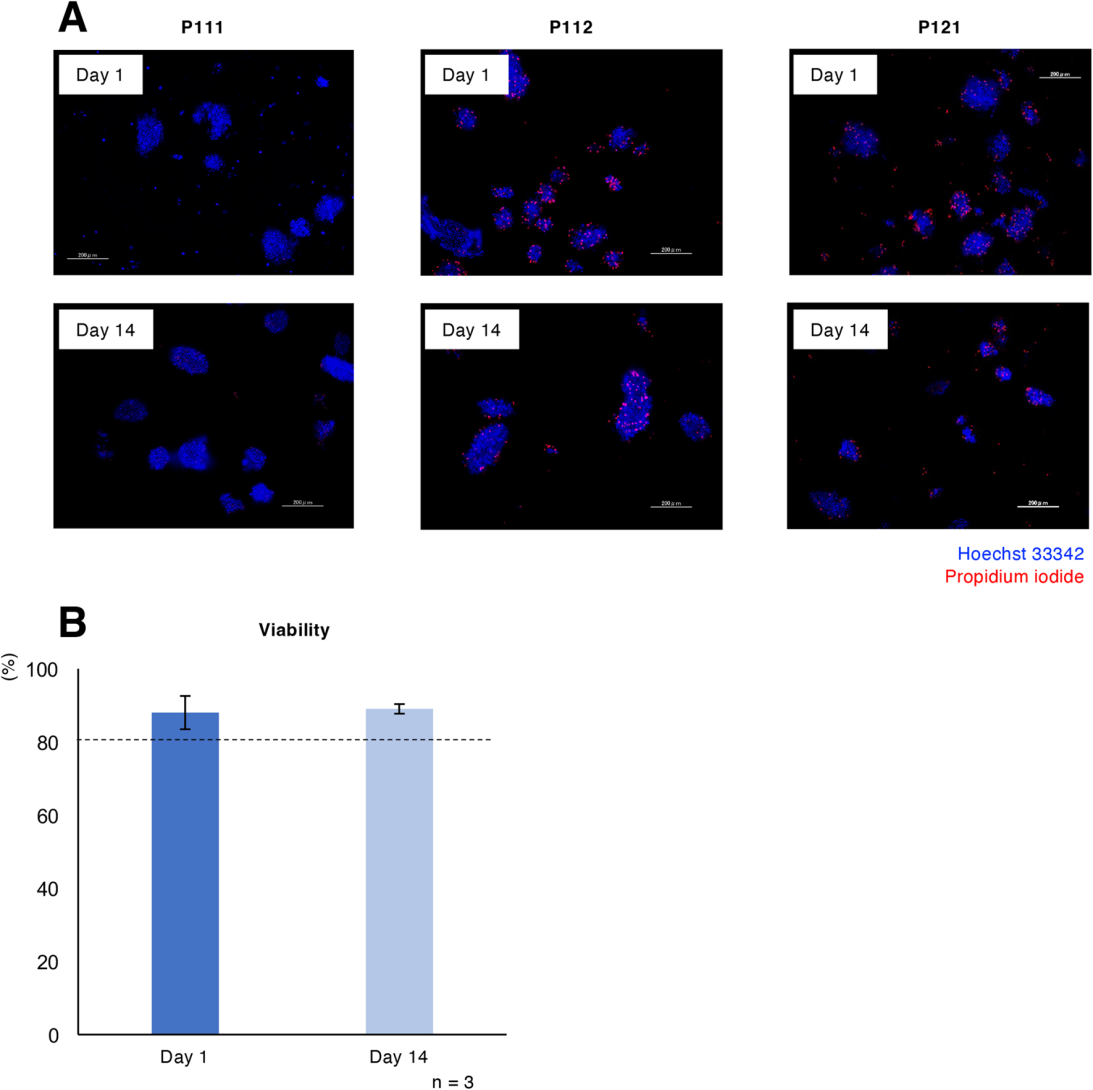
Viability of relatively short-term cultured porcine islets. (**A**) Hoechst 33342 (blue) and propidium iodide (red) double-stained porcine islets from pigs P111, P112 and P121 on days 1 (upper) and 14 (lower) of culture. (**B**) Average viability of the cultured islets on days 1 and 14 among the three isolations. Scale bar: 200 µm.

Viability was also maintained at a high level after relatively short-term culture. There was no difference between Day 1 and Day 14 (88.0% ± 4.6% vs. 89.0% ± 1.3%; Fig. 2A, B).

These data indicated that relatively short-term culture did not impair the morphology and viability of adult porcine islets.

### Relatively short-term culture preserved endocrine function of adult porcine islets, but genes correlated with endocrine hormone were impaired

**Figure 3:**
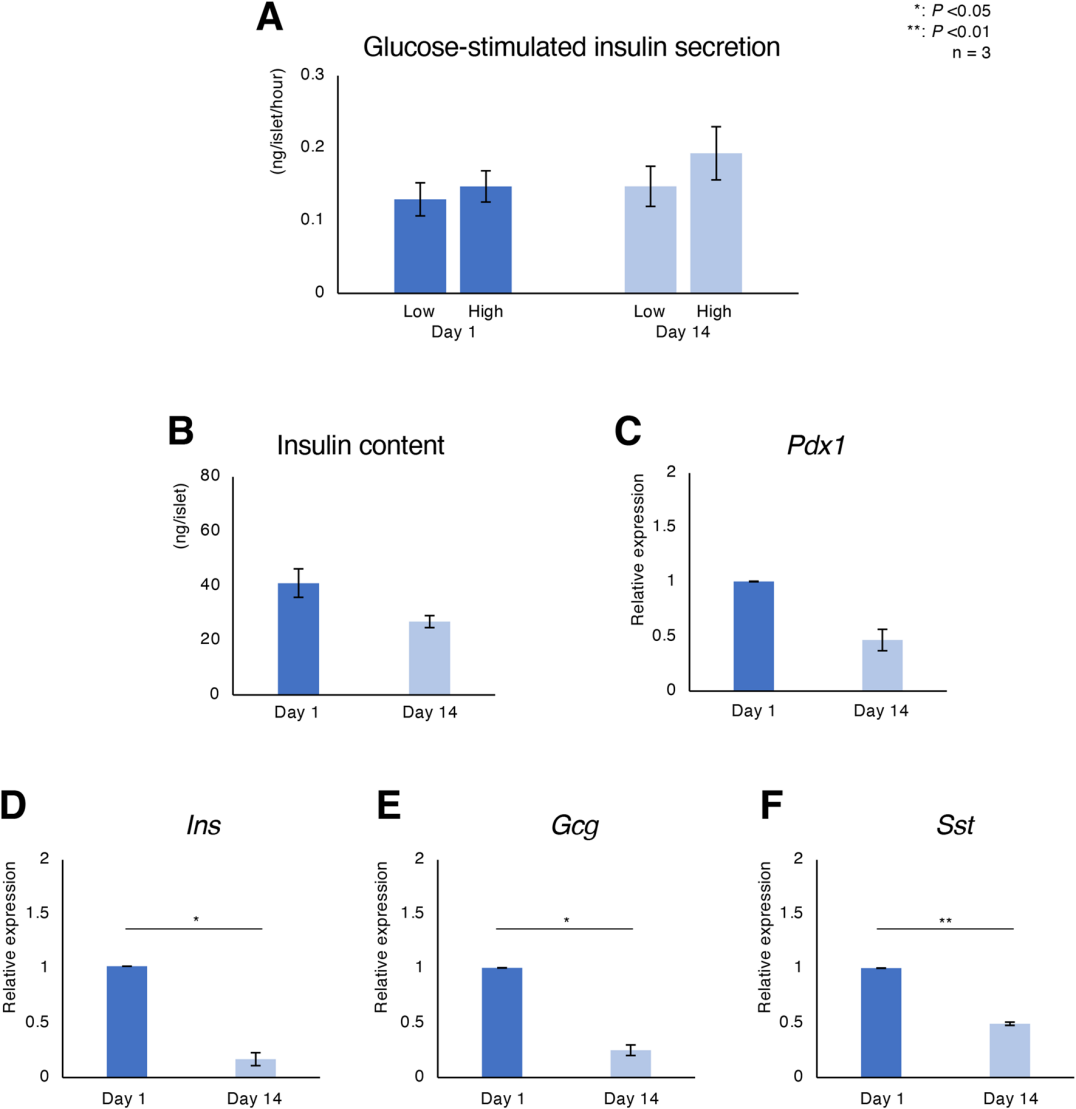
Endocrine function of relatively short-term cultured porcine islets. (**A**) Glucose-stimulated insulin secretion by cultured islets in response to low (3.3 mM) and high (16.5 mM) glucose concentrations on days 1 and 14 of culture. (**B**) Insulin content of cultured islets on days 1 and 14. (**C**)–(**F**) Expression of genes involved in pancreatic differentiation (**C** *Pdx1*) and endocrine hormones (**D** *Ins*, **E** *Gcg*, **F** *Sst*) in cultured porcine islets. Day 1: blue, Day 14: pale blue. Ratios of gene expression on days 1 or 14 are shown as 2^−ΔΔCt^ values. *P < 0.05, **P < 0.01.

Next, we evaluated the endocrine function of relatively short-term cultured porcine islets. There were no significant differences in both GSIS and insulin content of islets between Day 1 and day 14 (Low: 0.13 ± 0.02 ng/islet/hour vs. 0.15 ± 0.02 ng/islet/hour, High: 0.15 ± 0.02 ng/islet/hour vs. 0.19 ± 0.04 ng/islet/hour, insulin content: 41.03 ± 5.18 ng/islet vs. 26.94 ± 2.20 ng/islet; Fig. 3A,B). The stimulation index was almost similar between Day 1 and Day 14 (1.19 ± 0.06 vs. 1.30 ± 0.01).

We then measured the expression levels of genes correlated with endocrine function in the relatively short-term cultured islets for each isolation. In this assessment, *Pdx1*, *Ins*, *Gcg* and *Sst* were measured. *Pdx1* is expressed on multipotent progenitor cells in pancreatic bud. It is required in the earliest step of pancreatic formation. The expression is maintained till the differentiation into β-cells^12^. The expression levels of *Ins*, *Gcg* and *Sst* were lower on Day 14 (Fig. 3C–F). These data indicate that 1 to 14 days of culture was acceptable for preserving the endocrine function of porcine islets. However, the expression levels of genes correlated with endocrine hormones were attenuated during culture.

### Influences of relatively short-term culture in expressions of porcine-specific antigens

**Figure 4:**
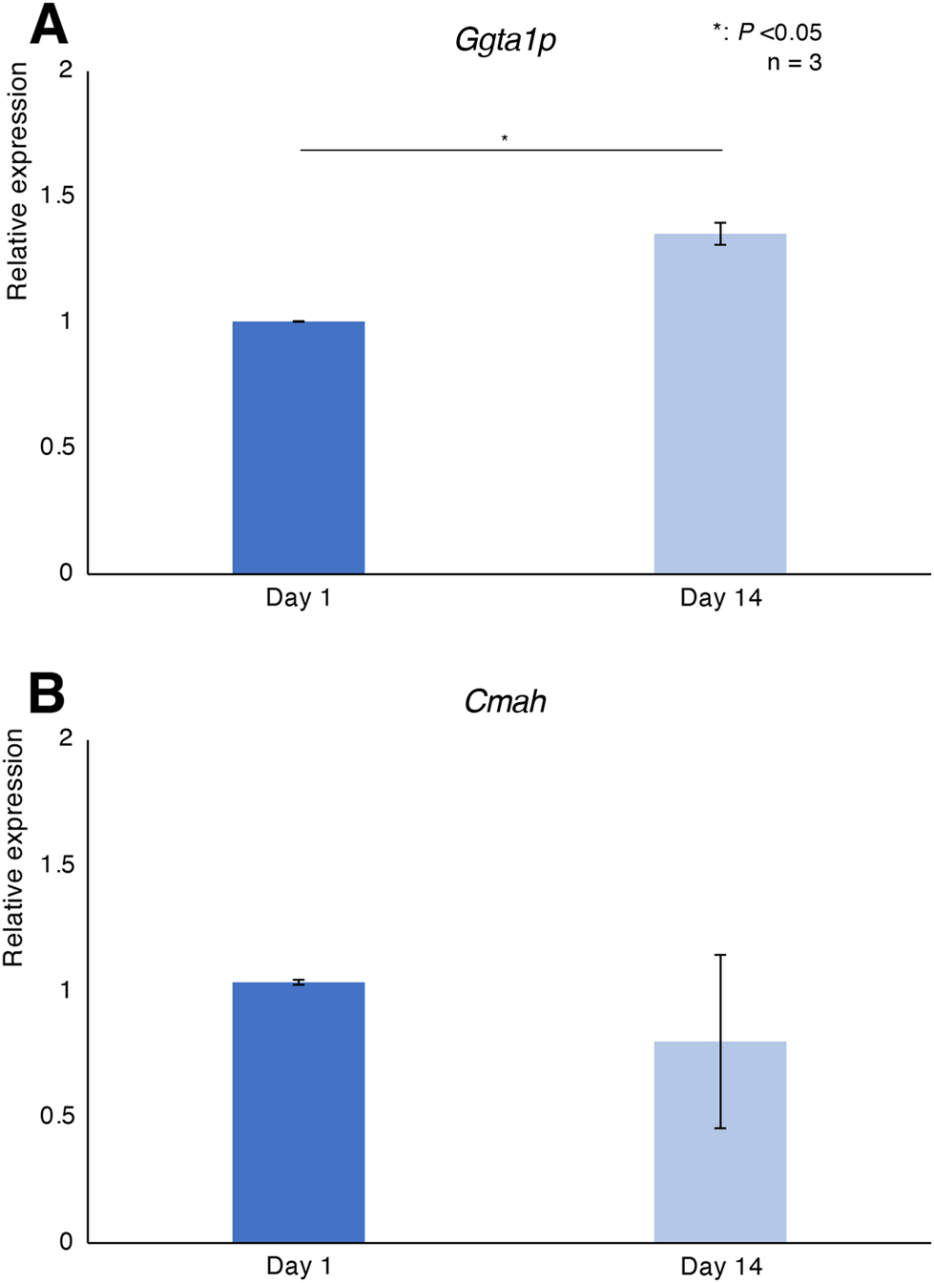
Porcine-specific antigens of relatively short-term cultured porcine islets. (**A**) and (**B**) Expression levels of genes correlated with porcine-specific carbohydrate antigen in cultured porcine islets (**A**: *Ggta1p*, **B**: *Cmah*). *P < 0.05.

Figure 4 shows the expression levels of genes correlated with porcine-specific antigens. The expression of *Cmah* (encoding cytidine monophospho-N-acetylneuraminic acid hydroxylase) was not changed on Day 14 (Fig. 4B). The expression of *Ggta1p* (encoding α-1,3-galactosyltransferase) of islets on Day 14 was higher than on Day 1 (1. 00 ± 0.00 vs. 1.35 ± 0.04, P < 0.05; Fig. 4A). We considered this increase was not prominent because 2^−ΔΔCt^ value was less than twice.

### Therapeutic effects of relatively short-term cultured porcine islet xenotransplantation were similar to overnight cultured islets

**Figure 5:**
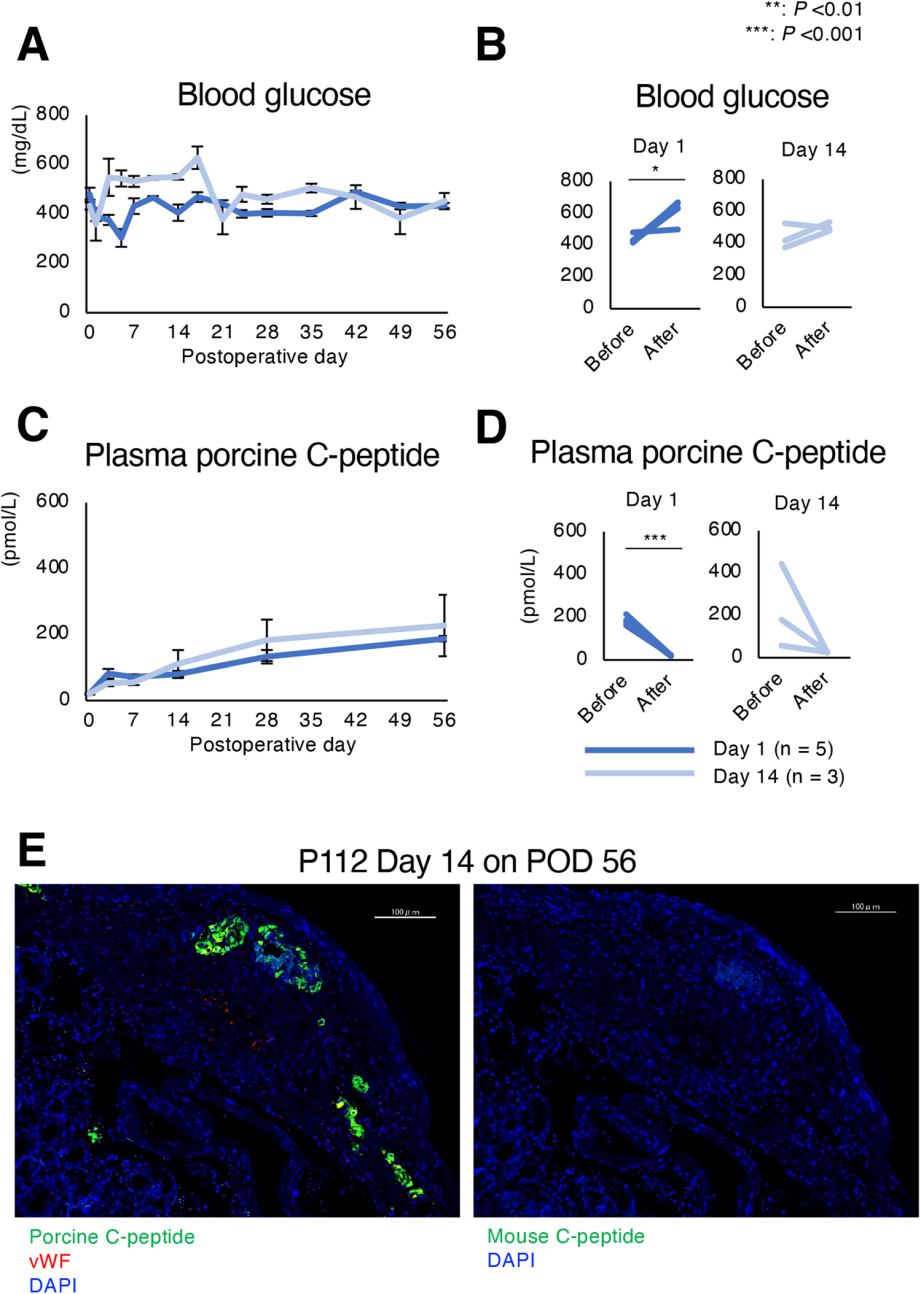
Xenotransplantation of relatively short-term cultured porcine islets. Effects of xenotransplantation of 2000 islet equivalents (IEQs), from pig P112, cultured at 24 °C for 1 day (Day 1: n = 5, blue) and 14 days (Day 16: n = 3, pale blue), into diabetic nude mice. (**A**) Blood glucose levels of recipient mice over 56 days. (**B**) Blood glucose levels of mice before and after graftectomy. (**C**) Plasma porcine C-peptide concentrations of recipient mice over 56 days. (**D**) Plasma C-peptide concentrations of mice before and after graftectomy. (**E**) Engrafted 14 day cultured islets on postoperative day (POD) 56. Islets were stained using anti-porcine C-peptide (green), anti-von Willebrand factor (vWF; red) (left) and anti-mouse C-peptide (green) antibodies. **P < 0.01, ***P < 0.001. Scale bar: 100 µm (**E**).

Finally, the effects of transplanting 2000 IEQs derived from relatively short-term cultured islets isolated from pig P112 into the renal subcapsular spaces of mice were assessed. There were no normoglycemic mice when islets on Day 1 or Day 14 were transplanted, but plasma concentrations of porcine C-peptide subsequently increased to similar levels in both groups (Fig. 5A,C). Blood glucose was reelevated and plasma porcine C-peptide disappeared plasma following graftectomy in both groups (Fig. 5B,D). Histological assessment revealed that the porcine islets were successfully engrafted 56 days following transplantation in both the Day 1 and Day 14 groups. The engrafted islets were positive for porcine C-peptide but negative for mouse C-peptide expression (Fig. 5E).

## Discussion

Porcine xenotransplantation is considered a feasible treatment option for the future, considering the success of two clinical trials with heart and kidney xenotransplantations using gene-edited pig tissue in 2022^13,14^. Regarding porcine islet xenotransplantation, a clinical trial using encapsulated technique was performed from 2009 to 2013 in New Zealand^15^. This trial revealed that encapsulated porcine islet xenotransplantation reduced unaware hypoglycemic events, and there was no detected infection from porcine endogenous retrovirus. Porcine islet xenotransplantation appears be to a reasonable strategy for promoting islet transplantation and treating severe diabetes. In principle, porcine islet transplantation can be done as required, because donor pigs can be prepared according to clinical needs. Therefore, elucidation of the acceptable culturing span of porcine islets is critical. Many studies have been carried out targeting the timespan of culture, however, more research is required. One group showed that porcine islets cultured for 11 days exhibited attenuated endocrine function^16^, and another group using rat recipients revealed that xenotransplantation of porcine islets cultured for 1.5–3.0 weeks engrafted for 5 months after transplantation^17^.

In this study, we assessed compared the characteristics of relatively short-term (14 days) cultured adult porcine islets, versus overnight cultured islets. It was revealed that relatively short-term culture preserved the morphology, viability, and function of porcine islets. Furthermore, the therapeutic effects of xenotransplantation, such as changes in plasma porcine C-peptide levels, were similar in relatively short-term and overnight cultured islets. These data indicated that relative short-term culture of porcine islets can be used for islet xenotransplantation. Regarding immunogenicity, the present study found that the expression of *Cmah* was not changed in relatively short-term cultures. Cytidine monophospho-N-acetylneuraminic acid hydroxylase transforms N-acetylneuraminic acid to Neu5Gc, one of the major porcine xenoantigens that can be targeted to achieve xenotransplantation, because humans produce anti-Neu5Gc antibodies^18^. The expression of *Ggta1p* was increased after relatively short-term culture, while it was not prominent. α-1,3-galactosyltransferase catalyzes the terminal step in the biosynthesis of α-galactosidase (α-Gal), a major target antigen for human and anti-pig antibodies^13^. α-Gal is also one of the major xenoantigens causing hyperacute rejection in pig–to–human xenotransplantation^19^, and may provide an essential target for the success of porcine organ xenotransplantation, noting α-Gal is expressed in porcine lung, heart, kidney and liver tissue^20–22^. Previous studies, and our recent study^11^, indicated that adult porcine islets have limited expression of α-Gal^23,24^, indicating α-Gal may not be a crucial target to eliminate for the success of xenotransplantation of porcine islets. Furthermore, the current progress of gene-edition technology enables to remove xenoantigens, including α-Gal, Neu5Gc and SDa, -knockout pig such as 10GE^25^.

While relatively short-term cultured porcine islets harbored acceptable quality in function as islets, levels of gene expression correlated with endocrine hormones were attenuated. The attenuation of gene expressions on Day 14 would occur the following attenuation of the hormone secretion after the day. Therefore, it is predicted that longer culture would impair endocrine function of the islets. Our previous study assessing long-term culture (28 days) of adult porcine islets found that the insulin-releasing function of long-term cultured islets was significantly damaged^11^. Therefore, modification of culture methods which prevents the damage is required, especially prevention of impairment of gene expression correlated with endocrine hormones. Assessment the precise time between promotion/suppression of insulin gene expression and production/attenuation of insulin might also be needed for this discussion. We predict that the attenuation of endocrine hormones correlated gene expression under relatively short-term culture and following endocrine function under long-term culture are due to oxidative stress. Bruni and colleague revealed that human islets cultured for 7 days exhibited apoptosis, which was inhibited by administration of antioxidant^26^. Therefore, removal of oxidative stress may be one of promising strategies for assuring the quality of cultured islets for transplantation procedures.

We have a major limitation in this study in transplantation model. We failed to succeed normoglycemia by porcine islet xenotransplantation using both islets on Day 1 and 14, in spite of detection of serum porcine C-peptide. This might be because of technical limitation and usage of only one batch of islet preparation (from P112). The other limitation is in the small sample size (n = 3) associated with large animal study.

In conclusion, we have assessed the influence of relatively short-term culture on characteristics of porcine islets for xenotransplantation. Our data showed that relatively short-term culture did not impair the quality of adult porcine islets in regard to function, morphology, and viability in vitro. Further improvement of the culture method which preventing impairment of gene correlated with endocrine hormone is warranted.

## Methods

### Study design

Isolated adult porcine islets were cultured in an incubator at 24 °C, 5% CO_2_, with medium changed every 2–3 days. Morphology, endocrine function, gene expression and viability of islets on day 1 and 14 of culturing (defined as Day 1 and Day 14, respectively) were assessed as described below. Islet xenotransplantation to diabetic nude mice was performed using these islets. The number of used islets for each examination on day 1 and 14 were based on the count on day 1 and 14, respectively.

### Study approval

The care of animals and experimental procedures complied with the principles of laboratory animal care [Guide for the Care and Use of Laboratory Animals, 8th edition (National Research Council, 2011)], and the experimental protocol was approved by the Animal Care and Use Committee of Fukuoka University (approval number: 2114119).

### Animals

Microminipigs (Fuji Micra Inc., Fujinomiya, Japan) weighing approximately 25–30 kg were used as donor animals and male BALB/c-nu mice (CLEA Japan Inc., Tokyo, Japan) aged 8–12 weeks were used as diabetic recipients. The animals were housed under specific pathogen-free conditions and had free access to food and water.

### Procurement of pancreata

Procurement of porcine pancreata was described previously^11,27^. Total pancreatectomy of donor pigs was performed for organ procurement under general anesthesia using isoflurane (Fujifilm Wako Pure Chemical Co., Osaka, Japan). After laparotomy, an Argyle Salem sump tube (Covidien Japan Inc., Tokyo, Japan) was inserted into the aorta, ligated in place and used for heparinization via intravenous injection of 400 IU heparin sodium/kg body weight (AY Pharmaceuticals Co., Tokyo, Japan). Subsequently, the pigs were exsanguinated by incising the vena cava in the thoracic cavity, and Belzer UW® Cold Storage Solution (Preservation Solutions, Inc. Elkhorn, WI, USA) was infused via the tube while the abdominal organs were cooled using crushed ice. After flushing of the circulation was completed, total pancreatomy was performed. An 18–24 gauge intravenous catheter (size according to the diameter of the pancreatic duct) was inserted into the pancreatic duct, and cold preservation solution (ET-Kyoto solution; Otsuka Pharmaceutical Factory, Inc., Naruto, Japan) containing 100 IU/mL ulinastatin (Mochida Pharmaceutical Co., Tokyo, Japan) was infused at 1 mL/g pancreas mass.

### Porcine islet isolation and purification

The porcine islet isolation method was also described previously^11,27^. A solution containing purified collagenase and protease [liberase MTF (0.5 g per 1 vial) and thermolysin (15 mg per 1 vial); Roche CustomBiotech, Penzberg, Germany] was instilled into the dissected pancreas via a catheter placed in the pancreatic duct. The distended pancreas was cut into several pieces and then placed into a Ricordi chamber. Tissue digestion commenced with gentle shaking of the Ricordi chamber with circulating warmed collagenase solution. After digestion, the tissue was diluted in RPMI solution containing 10% inactivated plasma and ulinastatin and then collected in Belzer UW® Cold Storage Solution. The purification process was performed using IBM 2991 (COBE 2991; Terumo BCT, Tokyo, Japan) and centrifugation with a continuous density gradient be-tween 1.077 and 1.100 g/cm^3^ created using Optiprep (Veritas Co., Tokyo, Japan). After centrifugation, gradient density solutions containing highly-purified islets (≥ 70%) were collected. Percentage purity was determined using the total number of cell clusters staining positive for dithizone (Sigma-Aldrich, St. Louis, MO, USA).

### Islet culture conditions

Islets were cultured in CMRL1066 solution (Corning, Corning, NY, USA) containing 5.5 mM glucose, 10% fetal bovine serum, 1% antibiotics and 200 U/L rapid insulin agent, under a 5% CO_2_ atmosphere. The islets were cultured at 24 °C overnight or for 14 days (Fig. 1). In this study, we applied 24 °C as a culturing temperature. 24 °C is known as a general temperature for culturing porcine islets. We showed some merits by culturing at 37 °C in long-term culture in morphological stability, islet proliferation and regeneration, and partial recovery of insulin-secretion in previous study. On the other hand, this culture condition had a demerit in losing cultured islets due to trapping by the islet-derived attached cells. Culturing at 24 °C prevented the proliferation of the attached cells and trapping islets by the cells. Information regarding the donor micro-minipigs and isolated islets is provided in Table 1.

### Assessment of islet number and viability

Islet number was assessed using two methods: counts and islet equivalents (IEQs). Islet number was defined as the number of cultured islets, and IEQ was defined as the number of 150 µm-diameter islets for the normalization of islet volume. Islet number, IEQ and the IEQ/islet number ratio, indicative of the mean size of the islets, were measured on Day 1. Calculation of IEQs was performed by the protocol shown in Integrated Islet Distribution Program (10.17504/protocols.io.bk5vky66).

Viability of cultured islets was measured on Day 1 and Day 14. To assess the viability of islets, isolated islets (100 IEQs/isolation) were stained with Hoechst® 33342 and propidium iodide (Thermo Fisher Scientific, Waltham, MA, USA) and the percentages of viable cells in each islet were calculated using the following formula:

$$\left( {{\text{Hoechst}}\textregistered {\text{ 33342 stained cells}}} \right){-}\left( {{\text{propidium}}\,{\text{iodide}}\,{\text{stained}}\,{\text{cells}}} \right)/{\text{Hoechst}}\textregistered \,{33342}\,{\text{stained}}\,{\text{cells}} \times {1}00\,\left( \% \right).$$

The accuracy of the viability was guaranteed by morphologically distinguishing islets and non-islets using labeled (Hoechst® 33342 and propidium iodide) and unlabeled islet images.

Image analysis was performed using ImageJ® software (National Institutes of Health, Bethesda, MD, USA).

The morphology of cultured islets was assessed using the islet quality score on Day 1 and Day 14. The assessment of the morphology was performed using dithizone-stained islets which were sampled for counting islets. Twenty to fifty islets were used for the assessment. Islet quality score was defined as the sums of the scores (0–10) for islet shape (flat/planar = 0, in between = 1, spherical = 2); border (irregular = 0, in between = 1, well-rounded = 2); integrity (fragmented = 0, in between = 1, solid/compact = 2); the number of single cells in culture medium (many = 0, a few = 1, almost none = 2); and diameters of the islets (all islets < 100 µm = 0, a few islets > 200 µm = 1, over 10% of islets > 200 µm = 2), as shown in Table 1^11^. This scoring was also following the protocol in Integrated Islet Distribution Program (10.17504/protocols.io.bk5vky66).

### Glucose-stimulated insulin secretion

The GSIS was measured using 300 IEQs. Islets were preincubated with 3.3 mM glucose for 60 min, after which they were stimulated with 3.3 mM (low) or 16.5 mM (high) glucose for 60 min using cell culture inserts (Millicell Hanging Cell Culture Insert, PET 8 µm, 24-well; Merck Millipore, Tokyo, Japan). The porcine insulin concentrations in culture media were measured using an ELISA (LBIS Porcine Insulin ELISA Kit; Fujifilm Wako Shibayagi Co., Shibukawa, Japan). The absorbances at 450 and 595 nm were determined using an iMark™ Microplate Absorbance Reader with Microplate Manager® Software 6 (Bio-Rad, Hercules, CA, USA). The stimulation index, the ratio of insulin concentrations generated under high- and low-glucose stimulation, was calculated.

### Measurement of insulin content

Insulin was extracted from 300 IEQs using 1 mL RIPA buffer (Nacalai Tesque, Kyoto, Japan) containing × 100 protease and phosphatase inhibitor cocktails (Nacalai Tesque). The insulin content was measured using an LBIS Porcine Insulin ELISA Kit.

### Real-time reverse transcription polymerase chain reaction (RT-PCR) analysis

RNA was extracted from 5000 IEQs porcine islets on Day 1 and Day 14 using TRIzol Reagent (Invitrogen) and purified using a PureLink® RNA Mini Kit (Thermo Fisher Scientific) according to manufacturers’ instructions. The mRNA concentrations were equalized using a NanoDrop 2000 spectrophotometer (Thermo Fisher Scientific). Reverse transcription was performed using a QuantiTect Reverse Transcription Kit (Qiagen K.K., Tokyo, Japan). Quantitative real-time RT-PCR analysis was performed using a CFX Connect Real-Time PCR Detection System (Bio-Rad Laboratories, Inc., Hercules, CA, USA) and a Thunderbird SYBR qPCR Mix (Toyobo Co., Ltd., Osaka, Japan). The primers used for real-time RT-PCR are shown in Table 2, and were designed by Fasmac Co., Ltd. (Atsugi, Japan). Relative quantitation was performed using LightCycler Software Version 4.1 and the results were normalized to the expression of a reference gene (*Actb*). Data are presented as a fold difference, calculated using the 2^−ΔΔCt^ method.

**Table 2:** Porcine primers for real-time reverse transcription-polymerase chain reaction analysis.

Primer name	Sequence (5′–3′)	Tm (°C)
*Actb*_F	CTCCAGAGCGCAAGTACTCC	60.18
*Actb*_R	TGCAGGTCCCGAGAGAATGA	60.61
*Ggta1*_F	GAAACCCAGAAGTTGGCAGC	59.68
*Ggta1*_R	CAGTCCACTAGCGGAAGCTC	60.18
*Cmah*_F	TCACATGCACTCAGACCACC	59.96
*Cmah*_R	CAACTGGACGCCACTCTGAT	60.04
*Ins*_F	GGCTTCTTCTACACGCCCAA	60.32
*Ins*_R	GCGGCCTAGTTGCAGTAGTT	60.39
*Gcg*_F	GATCATTCCCAGCTCCCCAG	59.89
*Gcg*_R	GTGTTCATCAGCCACTGCAC	59.76
*Sst*_F	CCCGACTCCGTCAGTTTCTG	60.39
*Sst*_R	GGCATCGTTCTCTGTCTGGT	59.75
*Pdx1*_F	AAGTCTACCAAGGCTCACGC	60.04
*Pdx1*_R	GCGCGGCCTAGAGATGTATT	60.04

### Induction of diabetes in recipient mice

Diabetes was induced in recipient mice by the intravenous injection of streptozotocin (220 mg/kg body weight; Sigma-Aldrich). Mice with blood glucose concentrations exceeding 400 mg/dL were used as diabetic recipients of islets.

### Islet transplantation

Recipient mice were anesthetized using isoflurane, then a dorsal incision was made through the muscle and peritoneum and the left kidney was mobilized outside the abdomen. The renal capsule was peeled off from the parenchyma to prepare the renal subcapsular space for the transplantation of islets. Two thousand IEQs porcine islets on Day 1 and Day 14 were placed into the space using Gastight Syringes (1002 RN; Hamilton Company Inc., Reno, NV, USA) and 0.58 mm Intramedic polyethylene tubing (Becton Dickinson, Franklin Lakes, NJ, USA). After transplantation, the kidney was returned to the abdomen and the incision was sutured.

### Assessment of the function of transplanted islets

The function of the transplanted islets was assessed by monitoring blood glucose and plasma porcine C-peptide concentrations, and changes in blood glucose concentration during glucose tolerance testing. Normoglycemia was defined as a blood glucose concentration < 200 mg/dL. Plasma porcine C-peptide concentrations were measured using a porcine C-peptide ELISA (Mercodia, Winston Salem, NC, USA).

### Histological assessment

The left kidneys of recipient mice were dissected following euthanasia and the transplanted islets were evaluated. Three-µm-thick sections were either stained with hematoxylin and eosin (HE) or subjected to immunohistochemistry (for insulin to identify islets, for von Willebrand factor (vWF) to identify vessels, for porcine C-peptide to identify porcine islets, and for mouse C-peptide to identify mouse islets. The primary antibodies used were mouse anti-pig C-peptide (1:200; Cloud-Clone Corp. MAA447Po21, Katy, TX, USA), mouse anti-mouse C-peptide (1:500; Novus Biologicals NBP1-05433, Centennial, CO, USA) and rabbit anti-vWF antibody (1:100; Abcam, Cambridge, UK). After incubation with primary antibody, donkey anti-mouse IgG (H + L) Alexa488 (1:100; Jackson ImmunoResearch Laboratories, Inc., West Grove, PA, USA) and Cy3-conjugated goat anti-rabbit (1:100; Jackson ImmunoResearch Laboratories, Inc.) were used as secondary antibodies. Nuclear staining was performed using 4′,6-diamidino-2-phenylindole (DAPI). Histological images were obtained using a BZ-X700 microscope (Keyence, Itasca, IL, USA).

### Statistical analysis

Blood glucose and plasma C-peptide concentrations were compared using two-way repeated measures analysis of variance, followed by Dunnett’s test, as appropriate. Paired t-test was used for other comparisons. Data are presented as the mean ± standard error of the mean. P < 0.05 was used to define statistical significance. All tests were two-sided. Statistical analyses were conducted using JMP®12.0.0 (SAS Institute Inc., Cary, NC, USA).

### Statement on ARRIVE guidelines

This study was reported in accordance with the ARRIVE guidelines. All experiments were performed in accordance with relevant guidelines and regulations.

## Data Availability

The datasets used and/or analyzed during the current study available from the corresponding author on reasonable request.
